# IDELVION: A Comprehensive Review of Clinical Trial and Real-World Data

**DOI:** 10.3390/jcm11041071

**Published:** 2022-02-18

**Authors:** Miguel Escobar, Maria Elisa Mancuso, Cedric Hermans, Cindy Leissinger, Wilfried Seifert, Yanyan Li, William McKeand, Johannes Oldenburg

**Affiliations:** 1 University of Texas Health Science Center, Houston, TX 77030, USA; 2 Center for Thrombosis and Hemorrhagic Diseases, IRCCS Humanitas Research Hospital, Rozzano, 20089 Milan, Italy; mariaelisa_mancuso@libero.it; 3 Haemostasis and Thrombosis Unit, Division of Haematology, Cliniques Universitaires Saint-Luc, Université Catholique de Louvain (UCLouvain), 1200 Brussels, Belgium; hermans.cedric@gmail.com; 4 Tulane University School of Medicine, New Orleans, LA 70112, USA; cleissi@tulane.edu; 5 CSL Behring, 35041 Marburg, Germany; wilfried.seifert@cslbehring.com; 6 CSL Behring, King of Prussia, PA 19406, USA; yanyan.li@cslbehring.com (Y.L.); william.mckeand@cslbehring.com (W.M.); 7 Institute of Experimental Haematology and Transfusion Medicine, University Clinic Bonn, 53127 Bonn, Germany; johannes.oldenburg@ukbonn.de

**Keywords:** hemophilia B, factor IX, extended half-life, annualized bleeding rates, albumin fusion protein

## Abstract

Hemophilia B is a bleeding disorder caused by a deficiency of coagulation factor IX (FIX). Treatment with FIX replacement products can increase FIX activity levels to minimize or prevent bleeding events. However, frequent dosing with standard-acting FIX products can create a high treatment burden. Long-acting products have been developed to maintain bleed protection with extended dosing intervals. Recombinant factor IX–albumin fusion protein (rIX-FP) is a long-acting product indicated for the treatment and prophylaxis of bleeding events and perioperative management in adult and pediatric patients. This review outlines data from all previously treated patients in the Prophylaxis and On-Demand Treatment using Longer Half-Life rIX-FP (PROLONG-9FP) clinical trial program and summarizes real-world data evaluating the use of rIX-FP in routine clinical practice. In the PROLONG-9FP program, rIX-FP demonstrated effective hemostasis in all patients at dose regimens of up to 21 days in patients aged ≥ 18 years and up to 14 days in patients aged < 12 years. rIX-FP has a favorable pharmacokinetic profile and an excellent safety and tolerability profile. Extended dosing intervals with rIX-FP led to high levels of adherence and reduced consumption compared with other FIX therapies. Data from real-world practice are encouraging and reflect the results of the clinical trials.

## 1. Introduction

Hemophilia B is a recessive congenital X-linked bleeding disorder caused by mutation in the *F9* gene, which results in a deficiency or absence of coagulation factor IX (FIX) [1]. Mutations in the *F9* gene may be inherited or occur spontaneously, with the disorder affecting around 3.75 per 100,000 males worldwide [1,2]. The lack of functional FIX protein causes reduced plasma FIX activity and can result in spontaneous bleeding or excessive bleeding following injury or surgery [1]. The frequency and severity of bleeding in patients with hemophilia B is generally correlated with the plasma levels of clotting factor compared with normal levels (mild: >5–40%; moderate: 1–5%; severe: <1%) [1,3].

In patients with hemophilia, bleeding into joints can lead to chronic pain and disability, while severe spontaneous bleeds, especially in the head or abdomen, can be life-threatening [1]. The primary aim of treatment for patients with hemophilia B is to use FIX replacement therapy to prevent or treat bleeding events. Prophylaxis is the gold-standard treatment for patients with a severe bleeding phenotype [1]. Standard-acting FIX replacement therapy products have a half-life of ~18–24 h and therefore require frequent administration, e.g., 2–3 times a week [1]. The need for frequent infusions can be a burden for patients and can lead to poor adherence, negatively impacting clinical outcomes [4]. Long-acting recombinant FIX (rFIX) products provide a therapy option that may improve patient adherence and patient health-related quality of life (HRQoL) [4].

rIX-FP (albutrepenonacog alfa, IDELVION^®^, CSL Behring) is a recombinant fusion protein that links rFIX with recombinant human albumin. Linking to albumin extends the half-life of rFIX to ~90–105 h due to the binding of albumin with neonatal Fc receptor (FcRn) in the body [5,6,7,8]. rIX-FP has demonstrated improved pharmacokinetics (PK) and pharmacodynamics with a considerably longer half-life than other rFIX and plasma-derived (pdFIX) products in preclinical and clinical studies [6,7,8,9,10,11]. In 2016, rIX-FP was approved by the European Medicines Agency (EMA), the United States Food and Drug Administration (FDA), and the Japanese Ministry of Health, Labor and Welfare for the treatment and prophylaxis of bleeding in adult/adolescent and pediatric patients with hemophilia B at dose intervals of 7 days; dose intervals of up to 14 days can be adopted in adult/adolescent patients whose disease is well controlled [12,13]. Extended dosing regimens with rIX-FP up to 21 days were approved by the EMA in 2020 for selected patients ≥18 years, provided they are well-controlled on a 14-day rIX-FP dosing interval [12].

This review outlines the pharmacokinetics, efficacy, and safety of rIX-FP in previously treated patients (PTP) as determined in the Prophylaxis and On-Demand Treatment using Longer Half-Life rIX-FP (PROLONG-9FP) clinical trial program and describes clinical outcomes and patient experience with rIX-FP in routine clinical practice.

## 2. The PROLONG-9FP Clinical Trial Program

**Figure 1:**
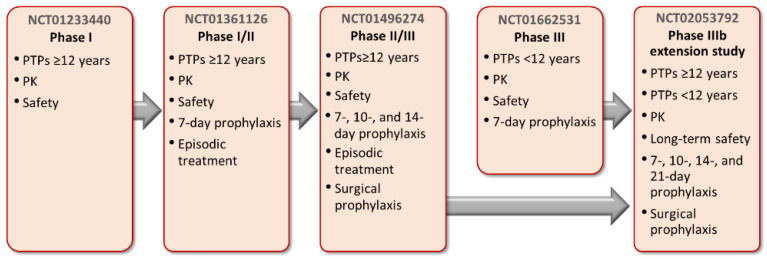
Overview of the PROLONG-9FP program. PK, pharmacokinetics; PTPs, previously treated patients.

The PROLONG-FP clinical trial program comprises five prospective, open-label, multicenter studies (NCT01233440, NCT01361126, NCT01496274, NCT01662531, NCT02053792) that assessed the use of rIX-FP in male patients with moderately severe hemophilia B (FIX ≤ 2%) (Figure 1) [6,7,8,11,14,15]. This review focuses on the results of the clinical trial program evaluating the PK profile, safety, and efficacy of rIX-FP in episodic and prophylaxis treatment regimens in previously treated patients (Figure 1). Studies were conducted in adult/adolescent (≥12–<65 years of age) and pediatric (<12 years of age) patient populations and included assessment of extended dosing regimens, management of surgical bleeding, and analysis of long-term outcomes. This review also discusses the real-world data and patient experience with rIX-FP beyond the clinical trials.

### 2.1. Pharmacokinetics

rIX-FP has undergone extensive PK profiling throughout the PROLONG-9FP program (Table 1). All PK analyses described in this section were calculated using plasma FIX levels determined with a validated one-stage clotting (OSC) assay using Pathromtin SL (Siemens Healthcare Diagnostics, Marburg, Germany) as an activator reagent [6]. In a phase I first-in-human dose-escalation trial in European adult/adolescent patients, rIX-FP had a mean terminal half-life of 92 h, as assessed after a single dose of 50 IU/kg. This was more than five times longer than the mean half-life of patients’ prior FIX therapy products (5.3 times higher than rFIX and 5.8 times higher than pdFIX) [6]. The mean incremental recovery of rIX-FP 50 IU/kg was 1.38 IU/dL per IU/kg, which is 46% and 25% higher compared with previous rFIX and pdFIX products, respectively [6]. The mean clearance of 0.75 mL/hour/kg of rIX-FP 50 IU/kg was 16% and 14% that of pdFIX and rFIX, respectively. A single dose of 50 IU/kg rIX-FP provided baseline-corrected trough levels of 13.4 IU/dL and 5.5 IU/dL at 7 and 14 days post-infusion, respectively [6]. Similar results were observed in a phase I/II prospective, open-label study in European adult/adolescent patients [15]. In 15 subjects who had not previously received rIX-FP, the mean terminal half-life was 94.8 h. The mean incremental recovery of rIX-FP 25 IU/kg was 1.52 IU/dL, and the mean baseline-uncorrected FIX activity at 7, 10, and 14 days was 5.6, 3.9, and 2.9 IU/dL respectively [15].

In a global phase III study in adult/adolescent patients, the mean terminal half-life of rIX-FP was 102 h, which was 4.3 times longer than that of prior FIX therapy [8]. A single IV dose of 25 IU/kg or 50 IU/kg rIX-FP maintained mean FIX activity > 5% up to day 10 and day 14 post-infusion, respectively [8]. Further analysis of data from this study demonstrated that 7- and 14-day rIX-FP prophylaxis regimens achieved consistently high steady-state trough FIX levels (22.3% and 12.5%, respectively), likely contributing to the observed median annualized spontaneous bleeding rate (AsBR) of 0 with both regimens (see below) [16].

In the long-term phase IIIb extension study, adult/adolescent patients whose disease was well controlled with rIX-FP on a 14-day regimen were eligible to receive rIX-FP every 21 days. Of these patients who wished to switch to a 21-day regimen, PK analysis after a single dose of rIX-FP 100 IU/kg was conducted. The reason for the differences in half-life and incremental recovery that was seen after 100 IU/kg compared with 25 or 50 IU/kg are unknown, but the authors of this study postulate that it may be due to increased distribution to the extravascular tissues and increased collagen-binding with the higher dose. Overall study results showed mean steady-state trough FIX activity levels of 22, 14, and 7.6% in patients on 7-, 14- and 21-day prophylactic regimens at median doses of approximately 50, 75, and 100 IU/kg, respectively [14].

In a global phase III study in pediatric (<12 years of age) patients, the mean terminal half-life of rIX-FP after a single dose of 50 IU/kg was 4.3 times longer than prior FIX therapy, at 91.4 h [7]. At the same dose, the mean clearance of rIX-FP was 1.11 mL/hour/kg, 6.4 times slower than prior FIX therapy. After a single 50 IU/kg dose, FIX activity was maintained >5% after 10 days and >2% after 14 days post-infusion [7]. During once-weekly prophylaxis with rIX-FP 35–50 IU/kg, the mean steady-state trough level was 12.8%. Further to this, long-term phase IIIb extension study data in pediatric patients showed that children < 12 years receiving rIX-FP 50–75 IU/kg every 14 days maintained a mean steady-state trough FIX level of 7.2% [11]. Overall, these results demonstrate a favorable PK profile for rIX-FP, showing that rIX-FP maintains FIX trough levels consistent with FIX levels seen in patients with a mild bleeding phenotype.

### 2.2. Population Pharmacokinetics

A population PK model for rIX-FP in patients with hemophilia B was developed based on 126 individual FIX activity level assessments from 104 adult/adolescent and pediatric patients with severe and moderately severe (≤2% FIX) hemophilia B who participated in phase III studies [17]. Simulation of a single dose of rIX-FP (25–75 IU/kg) predicted that the median trough FIX activity level would remain >5 IU/dL for up to 16, 12, and 9.5 days in adults/adolescents, older children (aged 6 to <12 years), and younger children (aged < 6 years), respectively. For steady-state dosing, the median trough FIX activity levels were predicted to be maintained at >5 IU/dL for the duration of the dosing interval for 25, 35, and 40 IU/kg weekly regimens and for 75 IU/kg every 14 days in adults/adolescents, and for 35 and 40 IU/kg weekly regimens in children. The population PK model correlated with clinical data, supporting dosing intervals of 7 and 14 days [17]. The model was updated to include data from the phase IIIb extension study and suggests that dosing intervals of 7 and 14 days in adults and children and up to 21 days in adults is sufficient to reach target FIX activity levels (>5%) [18]. Furthermore, rIX-FP may enable the majority of patients to transition from moderate/severe hemophilia B to mild disease with these dosing intervals.

### 2.3. Efficacy

rIX-FP has demonstrated efficacy for the episodic treatment of spontaneous and traumatic bleeds and for the prevention of bleeding events with routine prophylaxis or administered perioperatively. In a prospective phase II open-label study that assessed the safety and efficacy of rIX-FP weekly prophylaxis and episodic treatment in adult/adolescent patients, four patients treated episodically had a mean AsBR of 21.7, which was a 20% reduction in comparison to reported historical AsBR [15]. Thirteen patients receiving rIX-FP prophylaxis had a median AsBR of 1.1, which was ~50% lower than historical AsBR with prior prophylaxis for 10 of the 13 patients [15]. For the three patients who switched from episodic treatment to routine weekly prophylaxis with rIX-FP, a mean AsBR of 1.6 was observed, a 95% reduction of the historical mean AsBR of 31.7 [15].

The AsBR, total annualized bleeding rate (ABR), and annualized joint bleeding rate (AjBR) observed in patients receiving rIX-FP prophylaxis in phase III studies of the PROLONG 9-FP program are summarized in Table 2. A global phase III study in adult/adolescent patients evaluated the efficacy of 10-day and 14-day rIX-FP prophylaxis regimens for patients well controlled on a 7-day regimen, and the efficacy of rIX-FP weekly prophylaxis in patients switched from the episodic treatment of bleeding episodes with rIX-FP [8]. The median AsBR was 0.0 for patients receiving all prophylaxis regimens (n = 63), with similar data for ABR and AjBR across treatment regimens [8]. Nineteen patients who switched from rIX-FP episodic treatment to rIX-FP weekly prophylaxis experienced a 100% reduction in median AsBR (15.4 and 0.00, respectively; *p* < 0.0001) and a 90% reduction in median ABR (19.2 and 1.6, respectively; *p* < 0.0001). Of these 19 patients, 10 patients reported target joints during episodic treatment with rIX-FP, defined as at least three spontaneous bleeding episodes in the same joint in a 6-month period; on switching to prophylaxis 100% resolution of target joints was observed (*p* < 0.0001) [8]. An important finding of this study was that AsBR of the 14-day regimen was statistically comparable to the 7-day regimen, as determined by intrapatient analyses, demonstrating that, in selected patients, the efficacy of rIX-FP was maintained at extended dose regimens beyond every 7 days [8]. Patients treated episodically with rIX-FP had a slightly reduced ABR compared with the historical ABR prior to study entry (15.4 vs. 17.0, respectively). The hemostatic efficacy of rIX-FP was rated by investigators for the treatment of 358 bleeding events. For 94.2% of bleeding events, hemostatic efficacy was rated as excellent or good and 98.6% of bleeds were successfully treated with ≤2 infusions of rIX-FP [8].

In a phase III study of 27 pediatric patients receiving rIX-FP weekly prophylaxis, median AsBR was 0.0 for patients aged < 6 (n = 12) and 0.8 for patients aged ≥ 6 years (n = 15). ABR was 2.6 and 3.4, and AjBR was 0.5 and 1.1, for patients aged < 6 and ≥ 6 years, respectively. Most of the bleeding episodes reported, including joint bleeds, were due to trauma, which was reflective of the patient population [7]. With rIX-FP weekly prophylaxis, AsBR was reduced by 85–94% in 3 patients who were treated episodically prior to study entry. Three patients (aged < 6 years) had target joints prior to study entry, and target joints resolved in all patients during the study with rIX-FP weekly prophylaxis. Hemostatic efficacy of rIX-FP in the treatment of bleeding episodes was rated by investigators as excellent or good for 96.2% of bleeding episodes (a total of 106); 97.2% of bleeds were successfully treated with ≤2 infusions of rIX-FP [7].

A phase IIIb extension study that assessed the long-term safety and efficacy of rIX-FP in adult/adolescent and pediatric patients also evaluated extended rIX-FP prophylaxis dose regimens. Patients received 35–50 IU/kg every 7 days, or 50–75 IU/kg every 10 or 14 days. Additionally, patients ≥ 18 years who were well controlled on a 14-day regimen for ≥6 months could switch to a regimen of 100 IU/kg every 21 days. Results of the study showed that rIX-FP maintains good long-term prophylactic efficacy in patients aged ≥ 12 years with dosing intervals of 7-, 10- or 14 days and up to 21 days in selected patients aged ≥ 18 years [14]. Median AsBR of patients receiving 10- and 14-day dose regimens was 0.3 (n = 17) and 0.4 (n = 41), respectively, and intrapatient analyses showed that efficacy in the 14-day regimen was comparable to that of the 7-day regimen [14]. Median AsBR in patients ≥ 12 years of age receiving prophylaxis every 7 days (n = 22) and in patients ≥ 18 years of age receiving prophylaxis every 21 days (n = 11) was 0.00, with efficacy in the 21-day regimen comparable to the 7-day regimen by intrapatient analyses [14]. During the study, 8 of 59 patients developed target joints; at the end of the study target joints were resolved in 6 of 8 patients [14]. Overall, across all regimens, 98.0% of bleeds were treated successfully with ≤2 infusions of rIX-FP [14].

The long-term efficacy of rIX-FP and extended dose regimens in 24 patients < 12 years of age was also evaluated in the phase IIIb extension study. Patients could switch to 10- or 14-day dosing intervals if they were well controlled on a 7-day regimen for ≥6 months. Results showed very low median AsBRs across 7-, 10-, and 14-day regimens (0.0 [n = 21], 0.0 [n = 8], and 1.1 [n = 8], respectively), and intrapatient analyses showed that the 14-day prophylaxis regimen was comparable to the 7-day regimen in terms of AsBR [11]. On study completion, compared to the initial regimen, 4 (16.7%) patients were receiving rIX-FP with an extended dose regimen, 16 (66.6%) were on the same dose regimen, and 4 (16.7%) were receiving rIX-FP with a shortened dose regimen [11]. The proportion of bleeds requiring treatment that were successfully treated with ≤2 infusions of rIX-FP was 96% [11]

Overall, these studies demonstrated good efficacy of rIX-FP in patients of all ages with severe and moderately severe hemophilia B (FIX activity ≤ 2%) for both treatment and prevention of bleeding episodes using extended dose regimens for rIX-FP prophylaxis. In summary, in adult/adolescent patients, the efficacy of rIX-FP has been demonstrated for up to 14-day dose regimens, with the possibility of extending to 21 days in some patients aged ≥ 18 years, and in pediatric patients, efficacy has been demonstrated for a 7-day dose regimen, with the possibility of extending to a 10- or 14-day regimen in selected well-controlled subjects.

### 2.4. Consumption and Adherence

The extended dosing intervals achieved with rIX-FP can lead to reduced consumption of the drug compared with other rFIX products while maintaining efficacy. Phase III study data showed that consumption was decreased in patients of all ages treated with rIX-FP who switched from treatment with standard-acting FIX products regardless of dose regimen [19]. In adult/adolescent patients, mean monthly consumption was reduced by 37% compared with consumption of prior FIX treatment for both 7- and 10-day regimens and by 51% for the 14-day regimen (Table 2) (320.7 IU/kg previous FIX; 202.7, 201.5 and 157.4 IU/kg for 7-, 10- and 14-day rIX-FP regimens, respectively). The mean (SD) weekly dose of prior FIX was 69.9 (39.9) IU/kg and was 47.1 (10.9) with weekly rIX-FP; mean (SD) doses of rIX-FP for the 10- and 14-day regimens were 70.6 (9.6) and 71.9 (7.9) IU/kg, respectively. In pediatric patients, the mean weekly dose of prior FIX was 107.1 IU/kg vs. 47.2 IU/kg with weekly rIX-FP prophylaxis. Patients of all ages had a median AsBR of 0.0, demonstrating that a reduction in FIX consumption did not impact the efficacy of rIX-FP [19].

Long-term phase III study data have also shown that in patients of all ages, rIX-FP consumption is reduced for extended dosing intervals while bleed protection is maintained [11,14]. In adult/adolescent patients, mean (SD) monthly consumption was 206.4 (43.4), 212.3 (26.3), 158.0 (17.9) and 146.9 (5.5) IU/kg for the 7-, 10-, 14- and 21-day dose regimens, respectively (Table 2) [14]. In pediatric patients, mean (SD) monthly consumption was 231.2 (42.0), 224.2 (58.4) and 185.4 (24.0) IU/kg for the 7-, 10-, and 14-day prophylaxis regimens, respectively (Table 2) [11]. Low median AsBR was observed for all adult/adolescent and pediatric patients [11,14].

Patient adherence with prophylaxis treatment regimens is known to be essential for preventing bleeds in patients with hemophilia B. Adherence to prescribed prophylaxis regimens for patients of all ages and to prescribed dose in patients ≥ 12 years was evaluated in phase III clinical studies and in real-world practice. In clinical studies, 94.9% (56/59) of patients ≥ 12 years and 100% of 27 patients < 12 years received ≥80% of assigned prophylaxis regimen infusions [20]. The overall adherence rate to prophylaxis regimens was 95.5% across 7-, 10- and 14-day dose regimens in adult/adolescent patients, and was 97.9% in pediatric patients for a 7-day regimen. In patients ≥ 12 years of age, the overall adherence rate to dose was 91.1%, with 74.8% adherence rate to dose for patients treated episodically (n = 23). Overall, 85.7% (54/63) of patients on rIX-FP prophylaxis were dose adherent. Data from real-world practice were practice identified from 36 patients (26 adult/adolescent patients (≥12 years) and 10 pediatric patients (<12 years)) from three HTCs in Italy (n = 14), Germany (n = 15) and the US (n = 7). Overall, 92% of patients were adherent to rIX-FP treatment (100% in Italy, 100% in Germany and 57.1% in the US). Across all three centers, bleed rates or the number of bleeds whilst receiving rIX-FP remained low. In all of the above studies, adherence was defined as receiving ±10% of their prescribed dose ≥80% of the time [20].

### 2.5. Use in Surgery

**Figure 2:**
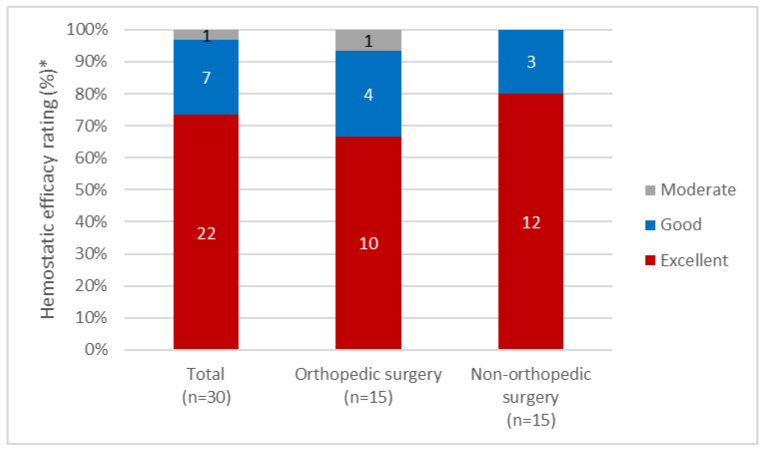
Hemostatic efficacy rating of rIX-FP in surgery. * Hemostatic efficacy ratings provided by the investigator/surgeon Figure adapted from Negrier, C.; et al. *Haemophilia* **2016**, *22*, e259–e266 and Pan-Petesch, B.; et al. *Thromb. Res.* **2020**, *193*, 139–141.

Patients with hemophilia B undergoing surgery are at increased risk of bleeds and are, therefore, treated with FIX therapy to prevent and minimize bleeding [21]. The efficacy and safety of rIX-FP in the perioperative setting was investigated as part of the phase III studies within the PROLONG-9FP clinical trial program [21,22,23]. In total, 30 surgeries in 21 adult/adolescent and pediatric patients have been reported. Prior to surgery, patients adhered to their normal prophylaxis regimen and the first preoperative dose was given approx. 3 h prior to surgery [22]. FIX activity levels were monitored before, during and after surgery to ensure target FIX levels based on the World Federation of Hemophilia (WFH) guidelines were achieved. Overall, the hemostatic efficacy of rIX-FP was rated as excellent or good in 93% of surgeries, of which 22 were major and 8 were minor (Figure 2). In total, 29/30 surgeries were managed successfully with a single preoperative bolus dose achieving a mean FIX activity level of 104.8 IU/dL, without additional intraoperative dosing. Mean (SD) overall consumption of rIX-FP in 30 surgeries was 250.1 (152.3) IU/kg, with preoperative and total postoperative consumption 74.9 and 125.6 IU/kg, respectively [22]. After minor surgery, 62.5% of patients (n = 5) did not require postoperative infusion of rIX-FP, and were able to resume their prophylaxis treatment 72 h postoperatively. Six joint-replacement surgeries in 5 patients required transfusions with re-bleeding occurring within 72 h of surgery; in these cases, significant blood loss was predicted prior to surgery. Patients undergoing surgery as part of the PROLONG-9FP trial did not experience treatment-related adverse events (TRAEs) and minimal postoperative complications were reported. No inhibitors, no antibodies for rIX-FP, or anaphylaxis have been reported [22].

### 2.6. Health-Related Quality of Life

Frequent FIX therapy infusions can burden patients with hemophilia B and impact HRQoL, which may in turn affect adherence and clinical outcomes. The efficacy and extended dosing of rIX-FP has been shown to translate into improved patient experience [24]. Patients < 12 years of age reported improved HRQoL with rIX-FP prophylaxis, particularly in the older pediatric patient age group (8–12 years) where improvements were seen in most domains of the Haemo-QoL questionnaire; in the younger age group, improvements were seen in “physical health”. Caregivers reported improved overall treatment satisfaction, with the biggest improvements seen in “burden” and “ease and convenience” [24]. With rFIX-FP prophylaxis, fewer patients and caregivers were affected by the patient having to miss school and ~95% of patients maintained physical activity levels [24].

### 2.7. Safety

A strong safety and tolerability profile for rIX-FP has been consistently demonstrated in phase I–III studies of the PROLONG-9FP program (Table 3) [7,8,11,14,15,21,23]. A low number of TRAEs have been reported in the clinical trial program (n = 15, Table 3); all TRAEs were rated as mild or moderate in severity and resolved on the same day without treatment [6,8]. In a phase IIIb extension study assessing the long-term safety of rIX-FP in adult/adolescent patients, the most frequently reported treatment-emergent adverse events (TEAEs) were arthralgia (25 events in 19 (32.2%) patients), headache (12 events in 6 (10.2%) patients), nasopharyngitis (10 events in 7 (11.9%) patients), and gastroenteritis (6 events in 6 (10.2%) patients) [14]. One of 16 treatment-emergent serious adverse events (SAEs) was considered to be treatment-related (peripheral ischemia) by the investigator. The sponsor considered the event to be more likely attributable to the surgical intervention of right total knee replacement followed by postoperative complications, including the entrapment of the tibiofibular artery in the healthy fibrosis around the knee implant [14]. The safety profile of the 21-day regimen in patients ≥ 18 years was comparable with the approved 14-day regimen in patients ≥ 12 years as no treatment-related TEAEs or SAEs were reported with either regimen and the majority of TEAEs were mild or moderate (96.6% with the 21-day regimen and 98.5% with the 14-day regimen) [14]. Phase IIIb extension study data for long-term safety of rIX-FP in pediatric patients showed that the most frequently reported TEAEs were pyrexia (15 events in 10 [41.7%] patients), nasopharyngitis (15 events in 8 (33.3%) patients), arthralgia (10 events in 6 (25%) patients), and headache (9 events in 5 (20.8%) patients) [11]. While 14 treatment-emergent SAEs were reported in 7 (29.1%) patients, none of these events were considered treatment-related [11].

In all phase I–III trials in PTPs, no inhibitors to FIX or antibodies to rIX-FP were observed after rIX-FP administration in previously-treated adult/adolescent and pediatric patients receiving rIX-FP prophylaxis and treated with rIX-FP episodically [6,7,8,11,14,15,21,22]. In addition, no anaphylactic reactions were reported (Table 3). There was one hypersensitivity reaction in the phase III trial with adult patients and the AE of hypersensitivity was considered likely to be an infusion-related reaction; all symptoms resolved within 23 min without treatment (Table 3). The patient chose to withdraw from the study and had no detectable inhibitors to FIX one month after treatment.

### 2.8. Clinical Study Data Summary

rIX-FP demonstrated a favorable PK profile, with a half-life considerably longer than standard-acting rFIX products, which contributes to long-term efficacy for the prevention of bleeds in routine prophylaxis and to hemostatic efficacy in the episodic treatment of bleeds. The efficacy of rIX-FP prophylaxis was maintained with extended dosing intervals while consumption was reduced. rIX-FP showed an excellent safety profile and no safety concerns were identified with extended dose regimens. The drug was well tolerated in previously treated adult/adolescent and pediatric patients with hemophilia B, leading to improved patient HRQoL and high adherence to rIX-FP regimens.

**Figure 3:**
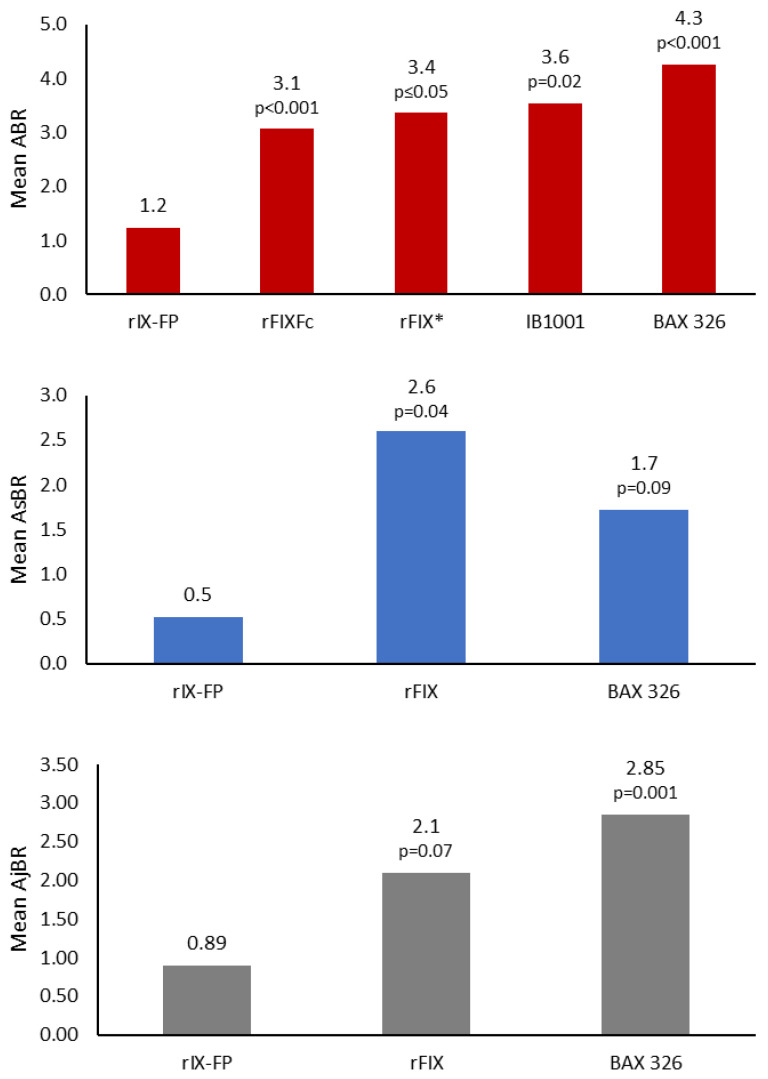
Efficacy of rIX-FP weekly prophylaxis compared with other rFIX products. * Mean ABR from Lambert et al.’s and Kavakli et al.’s studies. Figure adapted from Davis, J. et al., *J. Med. Econ.* **2019**, *22*, 1014–1021. Note, mean AsBR and mean AjBR data are not reported for rFIXFc or IB1001; mean ABR/AsBR/AjBR are not reported for N9-GP. Median doses for each product are as follow: rIX-FP, 40.3 IU/kg (7-day dosing) [8]; rFIXFc, 45 IU/kg (7-day dosing) [26]; rFIX, 100 IU/kg (7-day dosing) [27]; BAX 326, 50.5 IU/kg (2×/weekly dosing) [28]; IB1001, 53 IU/kg (2×/weekly dosing) [29]. ABR, annualized bleeding rate; AsBR, annualized spontaneous bleeding rate; AjBR, annualized joint bleeding rate; rFIX, recombinant factor IX.

Furthermore, while direct comparisons between products cannot be made, it has been observed that the prophylactic efficacy of rIX-FP is favorable over other standard half-life rFIX and extended half-life products [25]. A systematic literature review indirectly compared the efficacy of rFIX products in adult patients and showed that ABR with 7-day rIX-FP prophylaxis was significantly lower than with standard half-life (*p* ≤ 0.05) and other extended half-life (*p* < 0.001) rFIX products (Figure 3) [25].

## 3. Clinical Experience

In addition to data from PROLONG-9FP studies, real-world clinical data has been collated and analyzed to determine the effectiveness, safety, and clinical response of rIX-FP in routine clinical practice. Two post-marketing studies have used retrospective patient chart data to compare bleeding rates, FIX product consumption, and dosing regimens in adult/adolescent and pediatric patients with hemophilia B who switched from prior FIX prophylaxis to rIX-FP prophylaxis [30,31].

The first post-marketing study collected de-identified patient chart data from 24 sites in Germany and identified 81 patients treated with rIX-FP (54/81 (66.7%) severe hemophilia B; 27/81 (33.3%) mild/moderate hemophilia B) [30]. Of these 81 patients, 72 (89%) were treated prophylactically, including 59 (73%) who were also on prophylaxis with a prior product. For patients who switched from prior FIX prophylaxis to rIX-FP prophylaxis, and who had bleeding data available (42/59; 76% severe hemophilia; 24% mild/moderate hemophilia), mean (SD) ABR decreased from 2.6 (2.9) to 0.3 (0.6). The proportion of patients with zero bleeds increased by ~60% with rIX-FP vs. prior therapy (81 vs. 24%) [30]. For patients who received episodic treatment with prior therapy (n = 5), mean (SD) ABR was 5.3 (2.8), but when switched to rIX-FP prophylaxis, the mean (SD) ABR was 0.2 (0.4). Of the 59 patients previously on prophylaxis with standard half-life FIX, 66% were dosed at least twice a week, and 7% infused once a week or at a lower frequency. In contrast, 68% of the 72 patients on prophylaxis with rIX-FP were dosed every 7 days, and 22% of patients, who were well controlled, were dosed every 9 days or more. Mean weekly consumption decreased from 85.5 IU/kg/wk with nonacog alfa (n = 42) and 74.5 IU/kg with pdFIX products (n = 17) to 44.2 IU/kg/wk with rIX-FP (n = 72); this equates to a 48% decrease in consumption compared to nonacog alfa and a 41% decrease in consumption compared to pdFIX products [30].

The second post-marketing study identified 73 patients treated in 23 centers in Italy (n = 13), Belgium (n = 3) and the UK (n = 7) who switched to rIX-FP prophylaxis from prior FIX therapy [31]. Overall, 92.8% of patients had severe hemophilia B (Italy: 98%; Belgium: 70%; UK: 92%). The mean ABR for patients receiving rIX-FP vs. prior therapy was 0.2 vs. 3.5, 0.4 vs. 6.6 and 1.0 vs. 3.1 for patients in Italy (n = 44), Belgium (n = 7) and the UK (n = 22), respectively; this amounts to a 94.3%, 93.9% and 67.7% reduction in ABR, respectively. The proportion of patients with zero bleeds while receiving rIX-FP vs. prior therapy was 84.1 vs. 13.6%, 71.4 vs. 14.3%, and 36.4 vs. 0%, respectively [31]. Overall, for patients receiving prophylaxis, 9.6% of patients were dosed with prior FIX product once weekly, while after switching, 83.6% of patients were dosed with rIX-FP every 7–12 days, with 12.3 and 2.7% of patients on 14-day and 15-day dose regimens, respectively [31]. The mean weekly FIX consumption for patients switched to rIX-FP from prior FIX therapy was reduced 54, 71, and 59% in Italy, Belgium, and the UK, respectively [31].

Similar data showing lower ABR and reduced consumption with rIX-FP vs. prior rFIX therapy have been observed for patients treated on routine prophylaxis in the US [32]. The mean ABR (SD) for patients on rIX-FP was 0.7 (1.0) vs. 8.9 (9.6) on rFIXFc (n = 12) and was 1.5 (5.8) on rIX-FP vs. 4.5 (5.9) on nonacog alfa (n = 17) [32]. Mean (SD) weekly consumption with rIX-FP was 1.6 times lower than with rFIXFc (43.1 (17.5) vs. 67.4 (26.5) IU/kg, respectively; n = 37), and was 2.5 times lower than with nonacog alfa (47.1 (14.3) vs. 107.7 (38.4) IU/kg, respectively; n = 29) [32].

These data demonstrate the efficacy, reduced patient burden, and potential economic benefit of rIX-FP in clinical practice; however, retrospective chart review data may have some limitations [30,31,32]. These studies included a small number of patients and may not be representative of the entire patient population. In addition, no information was collected for the location, severity, treatment, and outcomes of bleeding events or for patient adherence. Generalization of results may not be applicable since only patients who switched from a prior therapy to rIX-FP were included in the analyses. Information was not provided for the reason why patients switched products and not all centers contributed all of their patient data, presenting the potential for patient selection bias. Factor consumption calculations were based on the most recent prescription of each product as an indication of likely stable dosing; however, dosing may have changed during treatment for optimization [30,31]. Regardless of potential limitations, the results of these real-world studies showed the effectiveness and usage data that were consistent with results observed in rIX-FP clinical studies of the PROLONG 9-FP program.

The safety profile and tolerability of rIX-FP in clinical practice have also been consistent with data shown in clinical studies [8,26,33,34]. One case of a patient with severe hemophilia B who developed a low-titer inhibitor during rIX-FP prophylaxis treatment has been reported [35]. The patient was switched to 50 IU/kg rIX-FP prophylaxis every 14 days from rFIX 30 IU/kg twice weekly to reduce the burden of frequent infusions. After effective treatment for 5 months, the patient developed spontaneous hematomas after 11 exposure days, and the presence of a low-titer inhibitor (0.9 BU/mL) was detected. The inhibitor was eliminated after 6 days’ treatment with rFIX (nonacog alpha) 30 IU/kg/bid, after which the patient was reverted to the previous rFIX prophylaxis regimen of 30 IU/kg twice-weekly [35].

### 3.1. Biodistribution

The biodistribution of rFIX products and the relevance of the extravascular space (EVS) are areas of interest related to the efficacy and PK profile of rIX-FP. The size of endogenous FIX allows for movement of the protein from the vasculature into the EVS, and the capability of rIX-FP to move into the EVS has been shown [17,36,37]. How a protein or drug distributes through the body can be represented by the mathematical PK terms volume of distribution (V_D_) and volume of distribution at steady state (V_SS_). A higher V_D_ or V_SS_ is observed for drugs that accumulate in extravascular sites, such as those that accumulate in adipose tissue or bind to extravascular or plasma proteins. Hence, a higher V_D_ or V_SS_ is potentially indicative of the extravascular distribution of a drug [38,39]. The V_SS_ of rIX-FP is almost that of natural pdFIX, at 0.923 dL/kg and 0.975 dL/kg, respectively [40].

There is limited evidence that extravascular FIX has a role in hemostasis in humans [37,40,41]. A clinical crossover study reported higher bleeding rates with rFIX 100 IU/kg once-weekly vs. 50 IU/kg twice-weekly dosing (ABR 4.6 vs. 2.6, respectively) despite similar recovery rates of the drug, suggesting that factors other than FIX PK impact bleeding potential [42]. Data from another clinical study assessing hemostatic efficacy of rFIX 100 IU/kg weekly in 25 patients showed that the three patients who had FIX trough levels of 0 reported an ABR of 0, suggesting that extravascular FIX may be contributing to hemostasis independent of circulating plasma FIX [27].

Currently, there is no conclusive evidence that extravascular FIX is associated with hemostasis or that FIX levels in the EVS translate to the clinical efficacy of rFIX therapy products. Some real-world experience studies have suggested that breakthrough bleeding in patients with hemophilia B receiving rIX-FP who have adequate factor trough levels may be due to the low extravascular distribution of rIX-FP. A retrospective chart review of adult/adolescent and pediatric patients with hemophilia B treated in select centers in the US reported higher spontaneous bleeding in patients receiving rIX-FP vs. other long-acting FIX products [43]. The cases of three adult patients treated in a single center in the US with no history of breakthrough bleeds on rFIX and experienced poor bleed control when switched to rIX-FP have also been reported [44]. It should, however, be noted that in all cases, patients were switched directly to a 14-day regimen with rIX-FP rather than demonstrating adequate bleed control on a 7-day regimen as recommended in the product label; two patients began a 14-day regimen with a dose of 60 IU/kg and the third patient had a dose of 65 IU/kg. Due to concerns for inadequate bleed control, all three patients switched to the recommended 7-day regimen and only one further breakthrough bleed was reported; two patients maintained the same dose when switching to the 7-day regimen (60 IU/kg and 65 IU/kg) and one patient reduced their dose (40 IU/kg) with no further bleeds [44]. These studies necessitate further investigations of FIX activity in relation to the EVS; despite the lack of clinical data on the distribution of rIX-FP, a study investigating the PK and whole-body distribution of rIX-FP in rats showed near-identical distributions of rIX-FP and rFIX in tissues throughout the body, including in joints [37,40].

### 3.2. rIX-FP in Patients with Mild/Moderate Hemophilia B

Mild hemophilia is defined as factor levels of >0.05–0.40 IU/mL (>5–40% of normal), and moderate hemophilia is defined as factor levels between 0.01–0.05 IU/mL (1–5% of normal) [1,45]. In contrast to severe hemophilia, which is often diagnosed early in life following spontaneous bleeding, mild/moderate hemophilia is usually diagnosed later in life following traumatic bleeding or excessive bleeding during surgery. There is a paucity of available research on mild/moderate hemophilia which is driven by patients with mild hemophilia often being ineligible to participate in clinical trials [45]. There are no established treatment regimens for patients with mild and moderate hemophilia to date. Patients with mild/moderate disease are less likely to receive prophylaxis treatment than those with severe disease, in whom it is considered standard of care [1,45,46]. However, real-world data show that ABRs are comparable between patients classed as having moderate and severe disease [1,46]. In patients with moderate hemophilia B, prophylaxis rather than episodic treatment may encourage patients to live a more active lifestyle by minimizing the increased risk of bleeds associated with trauma/injury, thus improving general health and quality of life for patients [4,47]. Clinicians should also remain aware of the potential discordance between disease severity class based on factor levels and bleeding phenotype, and consider patient bleeding history when recommending treatment options.

Females who have a deficient *F9* gene on one X chromosome (traditionally termed hemophilia B carriers) are likely to have <60% of normal FIX levels, and ~25% of carriers have factor levels < 40%, similar to patients with mild hemophilia B [48]. Carriers typically bleed more than women of the general population, with a higher degree of menorrhagia, prolonged bleeding after surgery, and postpartum bleeding reported [48]. Carriers with factor levels of <30% tend to be symptomatic and present with bleeding phenotypes akin to patients with mild hemophilia. However, carriers can have increased bleeding propensity despite normal factor levels [48]. It is recommended that hemophilia B carriers should be classed as female hemophilia patients and treated as such, receiving FIX replacement therapy as required [12,13,48]. Two cases of female hemophilia patients treated with rIX-FP have been reported from a single center in Belgium [49]; the first case was a pregnant 31-year-old woman with a mild bleeding phenotype (baseline FIX level before pregnancy, 0.16 IU/mL). The patient gave birth under epidural anesthesia following a single bolus of 6000 IU rIX-FP; her FIX level was 1.04 IU/mL post-bolus and remained at 0.35 IU/mL on Day 4. There were no delayed bleeding complications and no additional bolus was needed [49]. The second case was a 16-year-old girl with a severe bleeding phenotype (baseline FIX level, 0.01 IU/mL) who was previously treated with rFIX 2000 IU once weekly but was switched to rIX-FP 3000 IU every 2 weeks to reduce treatment burden and improve her quality of life. The patient’s FIX level increased from 0.09 IU/mL to 0.77 IU/mL following the first infusion of rIX-FP and remained at 0.10 IU/mL on Day 13 post-infusion. The patient has remained on prophylaxis with rIX-FP for over a year without any spontaneous bleeding events [49].

In patients with mild/moderate hemophilia B, treatment with a long-acting rFIX product such as rIX-FP would likely provide greater benefit than treatment with a standard-acting product since extended dose regimens may be less burdensome for patients and therefore more acceptable for those with mild bleeding phenotypes. Although this patient population does not experience spontaneous bleeding [45], prolonged bleeding may occur during surgery, therefore, the use of long-acting products would likely also be useful for perioperative management of mild/moderate patients; minimizing bleeding complications during surgery and allowing hemostatic control with few infusions. Indeed, several authors of this paper regularly use rIX-FP in this setting to improve patient management.

## 4. rIX-FP in the Clinic

### 4.1. Dosing

The recommended dose guidelines for rIX-FP in the most common clinical contexts for patients with hemophilia B are outlined in Table 4. The dose and duration of rIX-FP treatment are based on patient body weight, and are dependent on the severity of the FIX deficiency, the location and extent of bleeding, the patient’s age and clinical condition, and recovery of FIX [12,13]. The average observed recovery of rIX-FP in patients ≥ 12 years of age is 1.3 IU/dL, and in patients < 12 years of age is 1.0 IU/dL. For episodic treatment and perioperative management of bleeding, the required dose of rIX-FP is determined using the formula below, and the calculated required dose should be adjusted based on individual patient clinical condition and response [12,13].

- Required dose (IU) = body weight (kg) × desired factor IX rise (% of normal or IU/dL) × {reciprocal of recovery (IU/kg per IU/dL)}
  ○ Where the reciprocal of recovery is 0.77 for patients ≥ 12 years of age and 1.0 for patients < 12 years of age [12,13].
- Required increase in factor IX (IU/dL or % of normal) = dose (IU) × recovery (IU/dL per IU/kg)/body weight (kg)

The use of rIX-FP in surgery is aligned with WFH guidelines, which recommend that patients with hemophilia B have initial FIX activity levels of 60–100 IU/dL for major surgery and 50–80 IU/dL for minor surgery [1,12,13].

For routine long-term prophylaxis treatment, starting rIX-FP dose is typically between 25 and 50 IU/kg body weight every 7 days [12,13]. Prophylaxis dose should be adjusted based on individual patient response to the starting dose regimen. Shorter dosing intervals or higher doses may be required for some patients, particularly younger patients. rIX-FP dose guidelines vary slightly between Europe and the US, with FDA-approved doses for adult/adolescent patients being lower than those recommended by the EMA (Table 4) [12,13]. For patients of any age whose bleeding is well controlled on a once-weekly regimen, the dosing regimen may be extended to 50–75 IU/kg every 10–14 days [12,13]. For patients ≥ 18 years of age whose bleeding is well controlled on a 14-day regimen, a dose regimen of 100 IU/kg every 21 days can be considered [12,14].

For selected patients who are well-controlled on a 7-day dosing regimen with doses of 25–50 IU/kg, prophylaxis may be tailored through extended dosing intervals of up to 21 days in adults [14], or lower doses with the same dosing regimen. Real-world data from a single-center study in the UK showed that bleeding was effectively controlled in selected adult and pediatric patients with severe hemophilia B receiving a median dose of 20 IU/kg rIX-FP once-weekly; however, the observed median (interquartile range) ABR of 2 (1–6) was higher than that seen in clinical trials using a dose of 35–50 IU/kg. Trough levels and quality of life for patients receiving low-dose long-acting FIX products were comparable with prior standard-acting FIX therapy, and dosing frequency and factor consumption was reduced in patients receiving low-dose long-acting FIX vs. standard-acting FIX therapy [50]. This data, together with other data from routine clinical practice described above, suggests that there is potential to reduce patient burden and cost of treatment while maintaining effective bleed protection in patients with severe disease by switching from a standard-acting product to a long-acting product, such as rIX-FP [30,31,32,50]. However, it is important to note that while tailoring rIX-FP prophylaxis using extended dosing intervals or lower dosing may be an option in selected, well-controlled patients, this is not an option for all patients with severe hemophilia B. Examples of patient groups who may not be suitable for switching to extended dosing intervals include patients with high physical activity levels, who are at risk of traumatic bleeds, and patients who have lower FIX activity towards the end of the dosing interval.

### 4.2. Switching Patients to rIX-FP

While there is potential for any patient with hemophilia B to benefit from switching to rIX-FP prophylaxis from prophylaxis with standard-acting FIX therapy, certain patient populations or patients with specific characteristics are likely to benefit the most. These include pediatric patients, patients with recurrent joint bleeds (target joints), patients experiencing frequent breakthrough bleeds, patients with active lifestyles, patients with poor venous access, and patients with poor adherence to frequent treatment regimens [51]. Patients frequently treated episodically are also likely to benefit from prophylaxis with long-acting FIX therapy.

Patients considering switching should be given all the details of prophylaxis on rIX-FP and made fully aware of the potential benefits and risks of the therapy regimen ahead of making a final decision [51]. Before a patient can transition to prophylaxis with rIX-FP from prophylaxis with a standard-acting FIX therapy product or from episodic treatment, testing is recommended to assess rIX-FP PK in the individual [52]. Use of population PK models is recommended by the International Society of Thrombosis and Hemostasis (ISTH), and for rIX-FP, analyses may be performed using the Web Accessible Population Pharmacokinetic Service for Hemophilia (WAPPS-Hemo) [17,18,53,54,55].

Patients transitioning from prophylaxis with standard-acting FIX therapy or episodic treatment should be started on rIX-FP prophylaxis at the recommended dose on a once-weekly regimen (Table 4) [12,13]. Patients reporting well-controlled bleeding can consider treatment with extended dosing regimens [12,13]. Pediatric patients, patients with target joints, patients experiencing frequent breakthrough bleeds, and patients with active lifestyles may require shorter dosing intervals or higher doses [51]. Patient assessment is recommended after 10 exposure days on rIX-FP, as well as at 4-week and 3-month time points. Assessment of breakthrough bleeds and other clinical outcomes, as well as regular PK assessments to monitor FIX activity levels over time, and assessment of the quality of life and patient needs can direct adjustments of individual patient regimens for optimal treatment [51].

Another potential benefit of switching patients to long-acting products such as rIX-FP is cost. In a retrospective chart analysis, the estimated cost per success (defined as no spontaneous bleeding) for patients on rIX-FP compared to nonacog alfa was reduced by 29, 55, and 50% in Italy, Belgium, and the UK, respectively [31]. These data demonstrate the potential economic benefit of switching to rIX-FP in clinical practice; however, as previously noted, patient chart analysis may exhibit patient selection biases that may affect the generalizability of the results. Furthermore, calculating the true annual factor consumption cost of rIX-FP vs. prior FIX treatment in this analysis has some limitations, including that patients switched to rIX-FP from different products (rFIX, pdFIX) and list prices vary between countries and within hemophilia treatment centers [31]. Given these limitations, formal cost-effectiveness analyses are still needed.

### 4.3. Monitoring rIX-FP

For assessment of disease severity, PK profiling, and evaluation of changes in response to treatment, plasma FIX activity is regularly assessed in patients with hemophilia B. Measurement of FIX activity is most commonly performed using an OSC assay, which is the established method validated by the European Pharmacopoeia, and is used to assign potency labeling to rFIX products [56]. Interlaboratory and intralaboratory variability has been reported with the OSC [57,58,59,60], and it has been shown that the results of OSC assays for different rFIX products can be influenced by which surface activator compound is used as a reagent in the assay. The ISTH recommends the use of different reagents in OSC assays for different rFIX products to ensure the accuracy of rFIX activity measurement [61]. In a study comparing rIX-FP activity assessment using nine different activated partial thromboplastin time (APTT) reagents with Pathromtin SL as the standard, rIX-FP could be reliably measured with the majority of commercially available reagents; however, the use of actin FS or kaolin-based reagents consistently underestimated rIX-FP activity by ~50% when a plasma-derived standard was used for calibration [62]. This finding was observed both when samples were tested in a central laboratory and in a multicenter field study where locally prepared spiked samples were assessed using four different APTT reagents in five centers in four countries and centrally prepared spiked samples were assessed in eight centers in five countries. Furthermore, testing of paired samples in the central laboratory and 21 centers in 10 countries showed generally comparable results with 18 different APTT reagents except for actin FS and kaolin-based regents where substantial variability was demonstrated (±30% of central laboratory value) and the majority of samples were underestimated. Therefore, OSC assays that use APTT reagents are recommended for measuring rIX-FP activity [63].

## 5. Conclusions

In summary, a wealth of data on rIX-FP exists covering clinical studies and real-world use in various settings and populations. rIX-FP has been shown to provide effective hemostasis in adult/adolescent and pediatric patients with moderate-to-severe hemophilia B at dose regimens of up to 21 days in patients ≥ 18 years of age and up to 14 days in patients < 18 years of age, including during surgery. rIX-FP has a stable PK profile and an excellent safety and tolerability profile. Extended dose regimens with rIX-FP can reduce patient burden, and dose regimen flexibility allows for adjustment to optimize clinical outcomes and align with individual patient needs, potentially translating to increased patient adherence and improved HRQoL. Moreover, extended dose regimens result in reduced consumption of rIX-FP with potential economic benefit. Future studies may further explore extended dosing intervals and lower doses of rIX-FP to improve options for individualized dose regimens. The favorable efficacy and consumption of rIX-FP over standard-acting FIX therapy products and other long-acting FIX therapy products provide a basis for switching patients to rIX-FP. Data evaluating the use of rIX-FP in real-world clinical practice are encouraging and reflect results of the PROLONG-9FP clinical trials, showing that rIX-FP is well tolerated and effectively prevents and controls bleeding in previously treated patients with hemophilia B.

## Figures and Tables

**Table 1:** Pharmacokinetics of rIX-FP in PROLONG-9FP studies.

Single Dose rIX-FP	Patients ≥ 12 Years of Age				Patients < 12 Years of Age
	Phase I [6]	Phase II [15]	Phase III [8]	Phase IIIb Extension [14]	Phase III [7]
Dose (IU/kg)	50	25	50	100	50
Mean terminal half-life, hours	92	94.8	102	143	91
Mean FIX activity at 7 days, IU/dL	13.4 ^a^	5.6 ^b^	>5.0 ^b^	NR	>5.0 ^b^
Mean incremental recovery, IU/dL per IU/kg	1.38	1.52	1.27	1.02	1.01
Mean clearance, mL/h per kg	0.75	NR	0.77	0.66	1.11

^a^ Baseline-corrected. ^b^ Baseline-uncorrected. NR, not reported.

**Table 2:** Bleeding rates for patients treated with rIX-FP prophylaxis in phase III PROLONG-9FP studies.

rIX-FP Prophylaxis	Patients ≥ 12 Years of Age							Patients < 12 Years of Age			
	Phase III [8]			Phase IIIb Extension [14]				Phase III [7]	Phase IIIb Extension [11]		
Dose Regimen ^a^	7-Day	10-Day ^b^	14-Day ^b^	7-Day	10-Day	14-Day	21-Day ^c^	7-Day	7-Day ^d^	10-Day ^e^	14-Day ^e^
N	40	7	21	22	17	41	11	27	21	8	8
Median dose, IU/kg	40	75 ^f^	75 ^f^	49.7 (range: 19–90)	74.3 (range: 38–86)	74.9 (range: 7–106)	99.8 (range: 85, 111)	47.2 (IQR: 40.6–55.8)	49.0 (range: 22–86)	74.0 (range: 40–82)	73.7 (range: 56–82)
Mean consumption, IU/kg/month (SD)	202.7 (47.9)	201.5 (42.56)	157.4 (16.3)	206.4 (43.4)	212.3 (26.3)	158.0 (17.9)	146.9 (5.5)	205.1 (41.2) ^g,h^	231.2 (42.0) ^h^	224.2 (58.4) ^h^	185.4 (24.0) ^h^
Median AsBR (Q1, Q3)	0 (0, 0)	0 (0, 0)	0 (0, 1.0)	0 (0, 1.7)	0.3 (0, 1.1)	0.4 (0, 1.7)	0 (0, 0.5)	0 (0, 0.9)	0 (0, 0.5)	0 (0, 2.8)	1.1 (0, 3.4)
Median ABR (Q1, Q3)	0.0 (0, 1.9)	0 (0, 1.8)	1.1 (0, 2.7)	1.3 (0.4, 4.2)	0.8 (0.3, 4.9)	0.9 (0, 2.9)	0.3 (0, 2.5)	3.1 (0.9, 5.9)	2.0 (0.7, 4.7)	3.5 (0.8, 6.7)	5.6 (2.0, 6.9)
Median AjBR (Q1, Q3)	0 (0, 1.5)	0 (0, 0.9)	0 (0, 1.0)	0.8 (0, 2.3)	0.7 (0, 2.9)	0.1 (0, 2.3)	0 (0, 1.8)	1.0 (0, 2.3)	0.6 (0, 2.6)	2.0 (0, 3.8)	2.6 (0, 3.3)
Patients with zero spontaneous bleeds, n (%)	NR	NR	NR	10 (46)	9 (53)	18 (44)	7 (64)	14 (52)	14 (66.7)	5 (62.5)	3 (37.5)

^a^ Patients could be assigned to multiple regimens during the study. ^b^ Only patients with no spontaneous bleeds for ≥4 weeks receiving ≤40 IU/kg could switch to the 10- or 14-day regimen. ^c^ Only patients ≥ 18 years who were well controlled on a 14-day regimen for ≥6 months could switch to a 21-day regimen. ^d^ ABRs include only subjects who have been on each regimen for ≥12 weeks (n = 20). ^e^ Only patients who were well controlled on a 7-day regimen for ≥6 months could switch to a 10- or 14-day regimen. ^f^ Median assigned dose. ^g^ CSL Behring. Data on file, 2018. ^h^ Overall consumption, including prophylactic consumption and episodic consumption. ABR, annualized bleeding rate; AsBR, annualized spontaneous bleeding rate; AjBR, annualized joint bleeding rate; IQR, interquartile range; NR, not reported.

**Table 3:** Adverse event data for patients treated with rIX-FP in PROLONG-9FP studies.

Safety Data	Patients ≥ 12 Years of Age				Patients < 12 Years of Age	
	Phase I [6]	Phase II [15]	Phase III [8]	Phase IIIb Extension [14]	Phase III [7]	Phase IIIb Extension [11]
n	25	17	63	59	27	24
Mean EDs per patient	NR	51.5 ^a^	64.8	107 ^a^	61.9	155
Patients reporting TEAEs, n (%)	13 (52)	14 (82.4)	54 (85.7)	51 (86.4)	26 (96.3)	23 (95.8)
TEAEs, n	22	46	347	330	152	215
Mild, n	21	46	283	320	126	206
Moderate, n	1		59		23	
Severe, n	0	0	5	10	3	9
TRAEs, n	4	0	11	0	0	0
Mild, n	4	-	11	-	-	-
Moderate, n	0	-		-	-	-
Severe, n	0	-	0	-	-	-
Patients reporting TESAEs, n (%)	0	0	2 (3.2)	10 (16.9)	4 (14.8)	7 (29.1)
TESAEs, n	-	-	2	16	6	14
Mild, n	-	-	-	5	-	4
Moderate, n	-	-	-	5	-	3
Severe, n	-	-	-	6	-	7
TRSAEs, n	-	-	0	1	0	0
Patients who withdrew due to AE, n	0	0	1	1	0	0
Inhibitors or antibodies, n	0	0	0	0	0	0
Hypersensitivity, n	0	0	1	0	0	0
Injection site reactions, n (%)	1 (4.0)	NR	28 (0.7)	NR	48 (4.0)	NR
Thromboembolic events or anaphylactic reactions, n	0	NR	0	0	0	0

^a^ Patients on prophylaxis only. AE, adverse event; EDs, exposure days; NR, not reported; SAE, serious adverse event; TEAE, treatment-emergent adverse event; TESAEs, treatment-emergent serious adverse event; TRAE, treatment-related adverse event; TRSAE, treatment-related serious adverse event.

**Table 4:** Clinical treatment guidelines for rIX-FP.

Episodic Treatment and Perioperative Management of Bleeding		
Type of Bleed/Surgical Intervention	FIX Level Required (%) (IU/dL)	Frequency and Duration of Dosing
Minor or moderate hemorrhage	30–60	Single dose should be sufficient for most minor bleeds Maintenance dose after 24–72 h if bleeding does not cease
Major hemorrhage	60–100	Every 24–72 h for 7–14 days until bleeding ceases Maintenance dose weekly
Minor surgery	50–80	Single dose may be sufficient for most minor surgeries Maintenance dose after 24–72 h if bleeding does not cease
Major surgery	60–100	Every 24–72 h for 7–14 days until bleeding ceases Maintenance dose 1–2 times per week
Routine long-term prophylaxis		
Starting dose regimen	Patients ≥ 12 years of age	Patients < 12 years of age
EMA-recommended	35–50 IU/kg every 7 days	35–50 IU/kg every 7 days
FDA-approved	25–40 IU/kg every 7 days	40–55 IU/kg every 7 days
Patients of any age well-controlled on 7-day regimen	50–75 IU/kg every 14 days	

## Data Availability

This article is a review of previously published studies. CSL will only consider requests to share Individual Patient Data (IPD) that are received from systematic review groups or bona-fide researchers. CSL will not process or act on IPD requests until 12 months after article publication on a public website. An IPD request will not be considered by CSL unless the proposed research question seeks to answer a significant and unknown medical science or patient care question. Applicable country specific privacy and other laws and regulations will be considered and may prevent sharing of IPD. Requests for use of the IPD will be reviewed by an internal CSL review committee. If the request is approved, and the researcher agrees to the applicable terms and conditions in a data sharing agreement, IPD that has been appropriately anonymized will be made available. Supporting documents including study protocol and Statistical Analysis Plan will also be provided. For information on the process and requirements for submitting a voluntary data sharing request for IPD, please contact CSL at clinicaltrials@cslbehring.com.
